# Proteomic Analysis of Anti-inflammatory Effects of a Kampo (Japanese Herbal) Medicine “Shoseiryuto (Xiao-Qing-Long-Tang)” on Airway Inflammation in a Mouse Model

**DOI:** 10.1093/ecam/nep151

**Published:** 2011-03-10

**Authors:** Takayuki Nagai, Marino Nakao, Yuliko Shimizu, Yoshio Kodera, Masamichi Oh-Ishi, Tadakazu Maeda, Haruki Yamada

**Affiliations:** ^1^Kitasato Institute for Life Sciences and Graduate School of Infection Control Sciences, Japan; ^2^Oriental Medicine Research Center, Kitasato University, Tokyo, Japan; ^3^Laboratory of Biomolecular Dynamics, Department of Physics, Kitasato University School of Science, Sagamihara, Kanagawa, Japan

## Abstract

Effects of a Kampo (Japanese herbal) medicine “shoseiryuto (SST, xiao-qing-long-tang in Chinese)”, which has been used for the treatment of allergic bronchial asthma clinically, were examined on ovalbumin (OVA)-sensitized allergic airway inflammation model (i.e., bronchial asthma) in a mouse. When SST was orally administered at 0.5 g kg^−1^ day^−1^ from day 1 to 6 after OVA inhalation, SST reduced the inflammation in lung tissue, the number of eosinophils and the OVA-specific immunoglobulin E (IgE) antibody titer in bronchoalveolar lavage (BAL) fluids at 7 days after the OVA inhalation. SST also reduced the airway hyperreactivity at 6 days after the OVA inhalation. Proteomic analysis with the agarose two-dimensional electrophoresis showed that the expression of spectrin *α*2 was reduced in the lung tissue of OVA-sensitized mice and SST recovered the expression. Western blot and immunohistochemical analyses of lung tissue also confirmed this result. When prednisolone was orally administered at 3 mg kg^−1^ day^−1^ from day 1 to 6 after OVA inhalation, the inflammation in lung tissue, the number of eosinophils in BAL fluids and airway hyperreactivity were reduced in the OVA-sensitized mice. However, prednisolone did not reduce the OVA-specific IgE antibody titer in BAL fluids and did not recover the expression of spectrin *α*2 in lung tissue. These results suggest that at least a part of action mechanism of SST against OVA-sensitized allergic airway inflammation in a mouse model is different from that of prednisolone.

## 1. Introduction

Dramatic increases in the prevalence of bronchial asthma have occurred over the past few decades in Westernized countries and more recently in less-developed nations [1]. Estimates suggest that as many as 300 million persons are affected worldwide. Allergic bronchial asthma is characterized by elevated serum immunoglobulin E (IgE) antibody level and inflammation of the airways with recruitment of eosinophils in bronchoalveolar lavage (BAL) fluids [2–4]. Construction of airway smooth muscle and development of airway hyper-reactivity (AHR) are also hallmarks of bronchial asthma [4–6]. Shoseiryuto (SST, xiao-qing-long-tang in Chinese and so-cheong-ryong-tang in Korea) is a kind of Kampo (Japanese herbal) medicines and has been used clinically for the treatment of allergic rhinitis, bronchitis, bronchial asthma [7] and cold symptoms. *In vitro* antiallergic activities of SST include inhibition of histamine release and degranulation of mast cells [8, 9], proliferation of eosinophils [10], growth and differentiation of basophils [11], cholinergic effects for nasal gland acinar cells [12], synthesis of tumor necrosis factor *α* by peripheral blood mononuclear cells [13] and contraction of bronchial smooth muscle [14, 15]. *In vivo* antiallergic activities of SST include inhibition of passive cutaneous anaphylaxis (PCA) in rat [8, 16] and it showed efficacies in animal models of allergic rhinitis [17–19] and allergic asthma [20–22]. Previously, we have reported that SST reduced the productions of Th2 cell-associated cytokines, interleukin (IL)-4 and -5 [23, 24], and restored production of the Th1 cell-associated cytokine, interferon (IFN)-*γ* [23, 24], from lung CD4^+^ T cells and BAL fluid of ovalbumin (OVA)-sensitized allergic airway inflammation in a mouse model [25]. We also reported that anti-OVA IgE antibody levels were reduced in the BAL fluids of the sensitized mice after oral administration of SST [25]. However, the mode of actions of anti-inflammatory activity of SST against bronchial asthma remains to be fully elucidated.

In present study, we further elucidate the action mechanism of SST on the treatment of bronchial asthma. The *in vivo* effects of SST in mice were investigated by examining the AHR, recruitment of eosinophils and the production of allergen-specific IgE antibody, and compared with prednisolone using an OVA-sensitized allergic airway inflammation model. In present study, we have also adopted a proteomic analysis to identify lung protein(s) that are affected by SST treatment of the mouse model using agarose two-dimensional (2D) gel electrophoresis and mass spectrometry (MS)-based protein identification.

## 2. Methods

### 2.1. Materials

Eight medicinal plants were used for preparation of a Kampo medicine, SST. Pinelliae Tuber (tuber of *Pinellia ternata*) (lot no. TI302704, cultivated at Gansu Province in China in 2001), Ephedrae Herba (stem of *Ephedra sinica*) (lot no. 302423, cultivated at Inner Mongolia and Hebei Province in China in 1998 and 1999), Schizandrae Fructus (fruit of *Schisandra chinensis*) (lot no. 253109, cultivated at Jilin Province in China in 2000), Paeoniae Radix (root of *Paeonia lactiflora*) (lot no. YA302207, cultivated at Nara Prefecture in Japan in 2001), Glycyrrhizae Radix (root of *Glycyrrhiza uralensis*) (lot no. 253116, cultivated at Inner Mongolia in China in 2000) and Zingiberis Siccatum Rhizoma (scalded rhizoma of *Zingiber officinale*) (lot no. 302903, cultivated at Yunnan Province in China in 2001) were purchased from Uchida Wakan-yaku (Tokyo, Japan). Asiasari Radix (root of *Asiasarum heterotropoides* var. *mandshuricum*) (lot no. 20007151, cultivated at Gangwon Province in Korea in 2000) was obtained from Tsumura (Tokyo, Japan) and Cinnamomi Cortex (bark of *Cinnamomum cassia*) (lot no. 300801, cultivated in Vietnam in 2000) was from Tochimoto Tenkaido (Osaka, Japan). A voucher specimen of these plants was deposited at Laboratory of Biochemical Pharmacology for Phytomedicines, Kitasato Institute for Life Sciences, Kitasato University in Tokyo, Japan. SST extract was prepared as previously described [25, 26]. Briefly, a mixture of Pinelliae Tuber (6 g), Ephedrae Herba (3 g), Schizandrae Fructus (3 g), Cinnamomi Cortex (3 g), Paeoniae Radix (3 g), Asiasari Radix (3 g), Glycyrrhizae Radix (2 g) and Zingiberis Siccatum Rhizoma (1 g) was extracted with 600 mL of water. After the extract was centrifuged at 7000 × g for 30 min, the supernatant was collected and lyophilized (yield, 19.1%). Prednisolone sodium succinate was purchased from Shionogi & Co. Ltd. (Osaka, Japan). Rabbit anti-mouse spectrin *α*2 antibody and anti-*β*-actin monoclonal antibody were obtained from Santa Cruz Biotechnology (Santa Cruz, CA, USA) and Sigma-Aldrich (St. Louis, MO, USA), respectively.

### 2.2. Three-dimensional High Performance Liquid Chromatography Analysis of SST Extract

**Figure 1:**
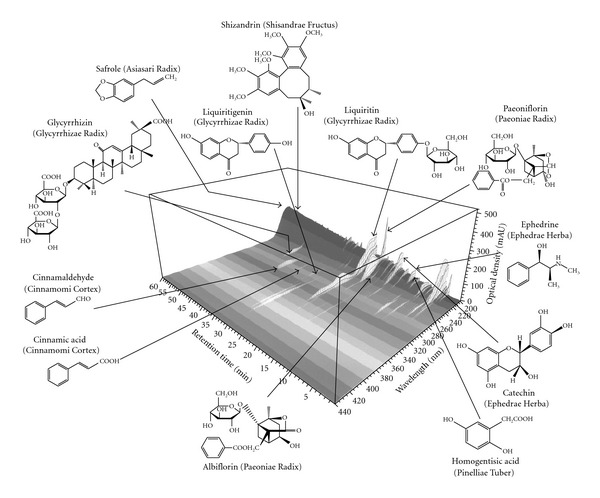
Chemical profile of the hot water extract from shoseiryuto as analyzed by 3D HPLC.

The three-dimensional (3D) high performance liquid chromatography (HPLC) analysis of SST extract was performed as previously described [25]. The 3D HPLC profile of an aqueous SST extract is shown in Figure 1. The analysis based on ultraviolet (UV)-absorption clearly showed the presence of the following major constituents in SST extract and the content of each constituent was shown in parenthesis: liquiritin (4.0 *μ*g mg^−1^ SST extract), liquiritigenin (1.5 *μ*g mg^−1^) and glycyrrhizin (25.1 *μ*g mg^−1^) (originating from Glycyrrhizae Radix); paeoniflorin (22.9 *μ*g mg^−1^) and albiflorin (12.6 *μ*g mg^−1^) (Paeoniae Radix); cinnamaldehyde (0.4 *μ*g mg^−1^) and cinnamic acid (1.7 *μ*g mg^−1^) (Cinnamomi Cortex); catechin (3.5 *μ*g mg^−1^) and ephedrine (12.9 *μ*g mg^−1^) (Ephedrae Herba); schizandrin (0.2 *μ*g mg^−1^) (Schisandrae Fructus); homogentisic acid (Pinelliae Tuber); safrole (Asiasari Radix). Contents of homogentisic acid and safrole in SST extract can not determined because each peak was overlapped with more than one constituent on the 3D HPLC profile.

### 2.3. Animals

Five-week-old female BALB/c mice were purchased from Japan SLC (Hamamatsu, Japan). The animals were housed in plastic cages in an air-conditioned room at 23  ±  2°C with a relative humidity of 55  ±  10% under a 12-h light-dark cycle, fed a standard laboratory diet and given water *ad libitum*. Animal experiments were approved by the Animal Research Committee of Kitasato University, and performed in accordance with the Guidelines for Care and Use of Laboratory Animals at Kitasato University and the National Research Council Guide for the Care and Use of Laboratory Animals in Japan.

### 2.4. Preparation of Allergic Airway Inflammation Model Mice

The mouse allergic airway inflammation model was prepared according to methods previously described [25–28]. Mice, 6-week-old, were sensitized by an intraperitoneal injection of 0.5 mL of alum-precipitated antigen containing 8 *μ*g of OVA (grade VI, Sigma-Aldrich) absorbed to 2 mg of alum (aluminium hydroxide hydrate gel, LSL) in saline vehicle. A booster injection of this alum-OVA mixture was given 5 days later. Non-sensitized control animals received alum only. Seven days after sensitization, the mice were exposed to provocation of antigen. For the challenge of antigen, the mice were placed in a chamber (Sanki Kagaku Kogei, Kawasaki, Japan) and were exposed twice to aerosolized OVA (0.5%) in phosphate buffered saline (PBS) for 1 h at 4 h intervals. The aerosolized OVA was produced by an ultrasonic nebulizer (Model NE-U12, OMRON, Tokyo, Japan).

### 2.5. Airway Responsiveness

Airway responsiveness was assessed with whole body plethysmograph (Buxco, Troy, NY, USA). This system estimates total pulmonary airflow using a dimensionless parameter known as enhanced pause (Penh). Six days after the challenge with aerosolized OVA, mice were placed in the chamber and exposed to methacholine chloride (an acetylcholine receptor agonist, Sigma-Aldrich) in saline for 3 min with nebulizer at increasing concentrations (3.125–50 mg mL^−1^) for dose responsiveness. Airway responsiveness was expressed as Penh value. Readings were taken for 5 min following each nebulization.

### 2.6. Specimens

Mice were anesthetized with diethyl ether and then bled from an axillary artery. Plasma was prepared from the blood with EDTA-2K as anticoagulant by centrifugation and used for titration of antibody. After bleeding, mice were incised ventrally along the median line from the xiphoid process to the point of the chin. The trachea with lung attached was taken out and washed twice by inflating the lung through the trachea with 2 mL of PBS containing 0.1% bovine serum albumin (BSA) and 1 mM EDTA-2K. The BAL fluid was used after the removal of cellular debris by centrifugation at 1200 × g for 20 min at 4°C. Eosinophils in blood and BAL fluid were stained with Hinkelman's solution and the number was counted using a hemocytometer, Fuchs-Rosenthal. Smear of blood or BAL cells on a glass slide was stained with a May-Grunwald Giemsa stain. Differential cell counting was performed using standard morphological criteria in which 100 cells were counted per slide.

### 2.7. Lung Histology

After preparation of BAL fluids, the right lung was inflated with 1 mL of 3.7% paraformaldehyde and then fixed in 3.7% paraformaldehyde for 2 days. After fixation, the right lung was embedded in paraffin with Tissue-Tek Infiltration Processor (Sakura Finetek Japan, Tokyo, Japan) and 2 *μ*m sections were cut with Sledge Microtome IVS-410 (Sakura Finetek Japan). Sections were then mounted on glass slide and stained with hematoxylin and eosin. Mucus production and goblet cell hyperplasia was examined by periodic acid-Schiff (PAS) staining.

### 2.8. Enzyme Linked Immunosorbent Assay for Anti-OVA IgE and IgG_1_ Antibodies

The OVA-specific IgE and IgG_1_ antibody titers in BAL fluid and plasma were measured by the modified fluorometric reverse (antibody-capture) enzyme linked immunosorbent assay (ELISA) [25, 29, 30]. Briefly, the wells of a 96-well ELISA plate (Immulon 4 HBX, Thermo Fisher Scientific, Rockford, IL, USA) were coated with 100 *μ*L of anti-mouse IgE or IgG_1_ mAb (BD Biosciences Pharmingen, San Diego, CA, USA; 1 *μ*g mL^−1^) in 50 mM carbonate-bicarbonate buffer (pH 9.5) containing BSA (10 *μ*g mL^−1^). The plate was incubated at 37°C for 3 h. After the solution was removed, the blocking solution, 1% skimmed milk (Morinaga Milk Industry, Tokyo, Japan) in PBS, was placed in each well (300 *μ*L) and incubated at 37°C for 1 h. The plates were washed four times with PBS containing 0.05% Tween 20 (PBS-Tween). Serial dilutions of BAL fluid or plasma with SuperBlock blocking buffer in PBS (Thermo Fisher Scientific) (diluted to 1 : 10 with PBS and added Tween 20 to 0.05%) were added to the wells (100 *μ*L). After being sealed with adhesive tape, the plates were incubated overnight at room temperature. After washing with PBS-Tween, biotinylated OVA (1 *μ*g mL^−1^) in the blocking solution was added to each well (100 *μ*L). The plates were incubated at room temperature for 1 h with shaking on a microplate mixer. After washing the wells, streptavidin-*β*-galactosidase conjugate (Calbiochem, EMD Chemicals, Gibbstown, NJ), diluted to 1 : 1000 with the blocking solution, was added (100 *μ*L) and incubated at room temperature for 1 h with shaking. After the final wash, 0.1 mM 4-methylumbelliferyl-*β*-d-galactoside (Sigma-Aldrich) in buffer A (10 mM sodium phosphate buffer, pH 7.0, containing 0.1 M NaCl, 1 mM MgCl_2_ and 0.1% BSA) was added to each well (100 *μ*L). The plates were sealed with tape and then incubated at 37°C for 2 h. The enzyme reaction was stopped by the addition of 100 *μ*L of 0.1 M glycine-NaOH (pH 10.3), and the fluorescence of the 4-methylumbelliferone measured (ex. 355 nm, em. 460 nm) using a Fluoroskan II spectrofluorometer (Thermo Fisher Scientific). Endpoint titers of OVA-specific IgE antibodies were expressed as reciprocal log_2_ titers.

### 2.9. Proteomic Analysis with Agarose 2D Electrophoresis

#### 2.9.1. Sample Preparation

The mice were sacrificed under light ether anesthesia, and the lungs were excised and stored at −80°C. Frozen tissues (*∼*20 mg) were cut into small pieces and disrupted using a Teflon glass homogenizer in 20 volumes of 7 M urea, 2 M thiourea, 2% w/v CHAPS, 0.1 M dithiothreitol, 2.5% w/v Pharmalyte pH 3–10 (GE Healthcare, Buckinghamshire, England) and protease inhibitors (Complete Mini EDTA-free; Roche Diagnostics, Mannheim, Germany), and then sonicated with Bioruptor UCD-200T (Cosmo Bio, Tokyo, Japan). The homogenate was centrifuged at 112 000 × g at 4°C for 30 min, and the clear supernatant was subjected to agarose 2D electrophoresis.

#### 2.9.2. Agarose 2D Electrophoresis

Two-dimensional electrophoresis was performed according to the procedure given by Oh-Ishi et al. [31–33]. Agarose gel was used for the first-dimension isoelectric focusing (IEF) and SDS-PAGE was for the second-dimension electrophoresis. The first-dimension agarose gel was 180 mm in length and 3.4 mm in diameter in a glass tube. The second-dimension polyacrylamide gel was 11% *T* homogeneous slab gel, and was 200 (width) × 120 (height) × 1.3 mm. Sample solutions (*∼*100 *μ*L) were subjected to the first-dimension IEF at 12 000 V h at 4°C, followed by fixation in 10% trichloroacetic acid and 5% sulfosalicylic acid for 1 h at room temperature (RT). After washing with water for 1 h, the second-dimensional SDS-PAGE was performed. Slab gels were stained with Coomassie Brilliant Blue (CBB, PhastGel Blue R-350, GE Healthcare) and destained in 30% v/v methanol containing 10% v/v acetic acid.

#### 2.9.3. Identification of Proteins Separated by 2D Electrophoresis

Protein separated by agarose 2D electrophoresis was identified by in-gel tryptic digestion of the protein followed by mass spectrometry (MS). Protein separated by agarose 2D electrophoresis was fragmented into peptides using an in-gel tryptic digestion. Protein spot in CBB stained 2D electrophoresis gel was excised in squares of *∼*1 mm *per* side, destained in 50% v/v acetonitrile (AcCN) containing 50 mM NH_4_HCO_3_ and then washed with deionized water. The gel pieces were dehydrated in 100% AcCN for 15 min and dried in a SpeedVac Evaporator (Waken-yaku, Kyoto, Japan) for 45 min. The pieces were rehydrated in 10–30 *μ*L of 25 mM Tris-HCl (pH 9.0) containing 25 ng *μ*L^−1^ trypsin (Sequencing Grade, Roche Diagnostics) for 45 min at 4°C. Excess trypsin was discarded and the gel pieces were incubated for 18 h at 37°C in a minimal volume (10–20 *μ*L) of 50 mM Tris-HCl (pH 9.0). Peptide fragments digested in gel pieces diffused into the surrounding solution over 18 h. The solution was slowly transferred to 1.5 ml siliconized plastic test tubes and stored at 4°C. Peptide fragments remaining in gel pieces were further recovered after 20 min incubations at RT in minimal volumes of 5% v/v formic acid containing 50% v/v AcCN. The solutions containing peptides were pooled in siliconized tubes at 4°C. Molar quantities of recovered peptide fragments were estimated from the staining intensity of the 2D electrophoresis spots that were digested in-gel with trypsin. Digested peptides equivalent to 10–20 pmol of protein in 2D electrophoresis spots were injected into a 30  ×  2.1 mm C8 RP column (Aquapore RP-300; Perkin Elmer, Norwalk, CT, USA) attached to a HPLC system (Nanospace SI-2; Shiseido Fine Chemicals, Tokyo, Japan). The column temperature was 45°C, and the flow rate of the mobile phase was 200 *μ*L min^−1^. The solvent composition of the mobile phase was programmed to change in 55 min cycles with varying mixing ratios (*r*) of solvent A (0.05% v/v formic acid) to solvent B (90% v/v AcCN and 0.05% v/v formic acid). The program for one cycle of a solvent composition change was as follows: constant mixing ratio *r* = 0% from time *t* = 0–5 min, linear gradient of *r* = 0–10% from *t* = 5–5.5 min, linear gradient of *r* = 10–50% from *t* = 5.5–30 min, linear gradient of *r* = 50–80% from *t* = 30–32 min, constant *r* = 80% from *t* = 32–36 min, downward linear gradient of *r* = 80–0% from *t* = 36–37 min and constant *r* = 0% from *t* = 37–55 min. Purified peptides were introduced from HPLC to an LCQ-DECA (ThermoQuest, San Jose, CA, USA), an ion trap mass spectrometer (ITMS), via an attached metal API2 needle (an ESI adapter). The MS and MS/MS peptide spectra were measured in a data dependent manner according to the manufacturer's operating specifications. SEQUEST was used to identify proteins from the MS and MS/MS spectra of peptides. This program searches entries in protein sequence databases by computing and reporting a SEQUEST score from the comparison. SEQUEST referred to the nr.Z and Swiss-Prot.Z protein sequence databases downloaded from ftp://ftp.ncbi.nih.gov/blast/db/. When the top ranked candidates had SEQUEST scores lower than 100 or when the top SEQUEST score was computed by using fewer than 10 peptide fragments, the raw MS and MS/MS spectra of peptides were inspected to judge their qualities. When the *m/z* ratios of the major peaks of the MS/MS spectra were corresponded to those of the *y*- or *b*-series of peaks calculated from the amino acid sequences of candidate peptides, the protein was identified.

### 2.10. Western Blotting

Expression of protein was determined by immunoblotting as previously described [34] with minor modification. In brief, the left lung was cut into small pieces and disrupted using a Teflon glass homogenizer in 20 volumes of 7 M urea, 2 M thiourea, 2% w/v CHAPS, 0.1 M DTT, 2.5% w/v Pharmalyte pH 3–10 and Complete Mini EDTA-free, and then sonicated for 10 s. The homogenate was centrifuged at 112 000 × g at 4°C for 30 min, and the clear supernatant was mixed with SDS sample buffer, boiled for 5 min and subjected to SDS-PAGE. Equal amounts of lysate protein were run on a 7.5% SDS polyacrylamide gel and transferred to nitrocellulose membranes (Hybond-ECL, GE Healthcare). Nitrocellulose blots were blocked with 1% BSA (Blot Qualified, Promega Corp., Madison, WI, USA) in Tris-buffered saline (TBS), and then incubated with anti-mouse spectrin *α*2 (1 : 200) and anti-*β*-actin (1 : 5000) in TBS containing 1% BSA. After washing with TBS containing 0.1% Triton X-100 (TBST), the blots were incubated for 1 h with horseradish peroxidase (HRP)-conjugated anti-rabbit IgG (Cell Signaling Technology, Beverly, MA, USA) at a dilution of 1 : 2000. Immunoreactive spectrin *α*2 protein was detected with the enhanced chemiluminescent protocol (Perkin Elmer Life Sciences, Waltham, MA, USA).

### 2.11. Immunohistochemistry

For spectrin *α*2 immunohistochemistry, lung sections on glass slides (MAS-GP type A) were deparaffinized and washed with water, followed by incubation in 3% H_2_O_2_/80% methanol for 20 min at RT to remove the endogenous peroxidase. After washing in PBS containing 0.3% Triton X-100 (PBS-T), sections were blocked for 3 h with 1% BSA in PBS-T, and then incubated for overnight at 4°C with rabbit anti-mouse spectrin *α*2 antibody (1 : 200). The sections were subsequently rinsed in PBS-T, incubated for 3 h at RT with biotinylated donkey anti-rabbit IgG (1 : 200, Molecular Probes, Eugene, OR, USA), and incubated for 3 h at RT with avidin-biotin complex using the ABC kit (Vector Laboratories, Burlingame, CA, USA). Spectrin *α*2-positive cells were visualized by incubating sections with diaminobenzidine (DAB) solution (Vector DAB, Vector Laboratories) as the chromogen, which results in a brown reaction product. Sections were dehydrated, penetrated and coverslipped using Permount (Fisher Scientific International Inc., Fair Lawn, NJ, USA).

### 2.12. Statistical Analysis

All data were expressed as the mean  ±  standard error of the mean (SEM). Results were analyzed by one-way analysis of variance (ANOVA). Post hoc comparisons, if applicable, were carried out using Fisher's Protected Least Significant Difference (PLSD), Dunnett's or Tukey's tests. *P*-values <.05 (*P* < .05) were considered indicative of significance.

## 3. Results

### 3.1. Histochemical Analysis of Efficacy of SST on Lung Inflammation

**Figure 2:**
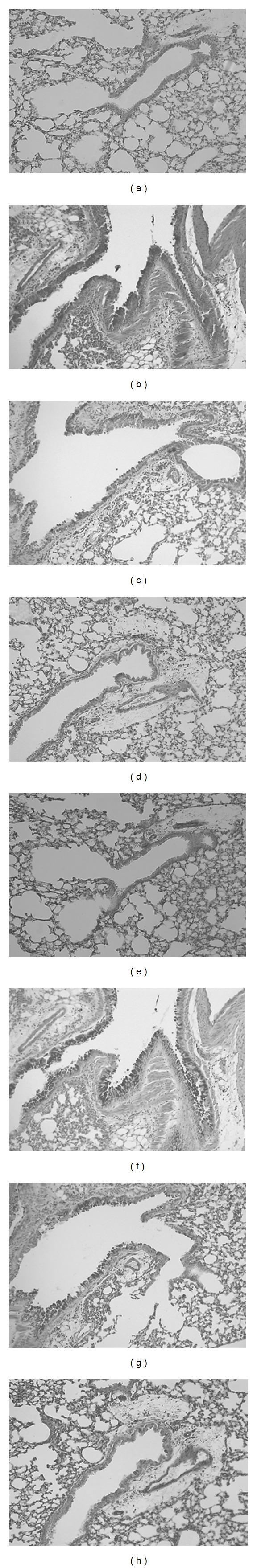
Effects of SST and prednisolone on lung tissue eosinophilia, hypertrophied smooth muscle and mucus secretion in OVA-sensitized mice were measured. Non-sensitized BALB/c mice were treated p.o. with water (a and e), and OVA-sensitized mice were treated p.o. with water (b and f), SST (0.5 g kg^−1^) (c and g) or prednisolone (3 mg kg^−1^) (d and h) five times from day 1 to 6 after OVA exposure. Lung tissues were removed at 7 days after OVA exposure and stained with hematoxylin and eosin (a–d) or periodic acid-Schiff (e–h) as described in “Methods” section. Magnification ×100.

OVA-sensitization induced marked infiltration of inflammatory cells, especially eosinophils, into the lamina propria, and perivascular and peribronchiolar areas as compared to non-sensitized and OVA-challenged control mice (Figures 2(a) and 2(b)). Oral administration of SST or prednisolone each attenuated the eosinophil-rich leukocyte infiltration compared with vehicle control (Figures 2(c) and 2(d)). On the other hand, OVA-sensitized and OVA-challenged mice, but not non-sensitized and OVA-challenged mice developed marked goblet cell hyperplasia and mucus hypersecretion within the bronchi in the lung (Figures 2(e) and 2(f)). The OVA-induced mucus secretion was significantly abated by SST and prednisolone compared to vehicle control (Figures 2(g) and 2(h)). These results suggest that SST and prednisolone decrease the inflammation in the lung of the OVA-sensitized and OVA-challenged mice on histochemical analysis.

### 3.2. Reduction of the Number and Percentage of Eosinophils by SST

**Figure 3:**
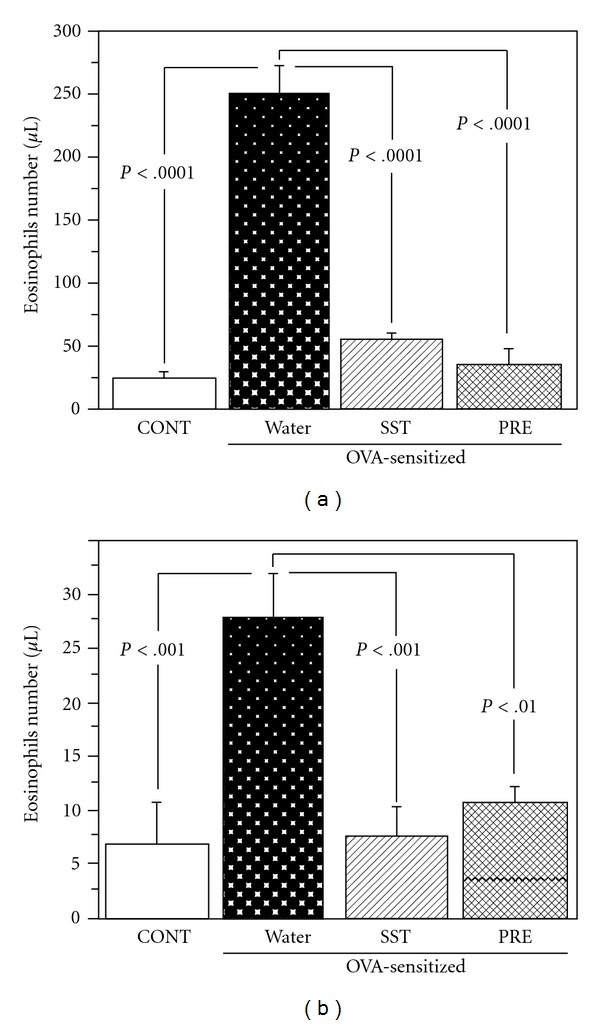
Effects of SST and prednisolone on number of eosinophils in blood and BAL fluid of OVA-sensitized mice were measured. OVA-sensitized mice were treated with SST, prednisolone (PRE) or water as described in legend of Figure 2. Number of eosinophils in blood (a) and BAL fluid (b) were counted on 7 days after the OVA exposure. Each column represents the mean  ±  SEM of five to seven mice per group. Statistical analysis was conducted using Tukey's test.

**Figure 4:**
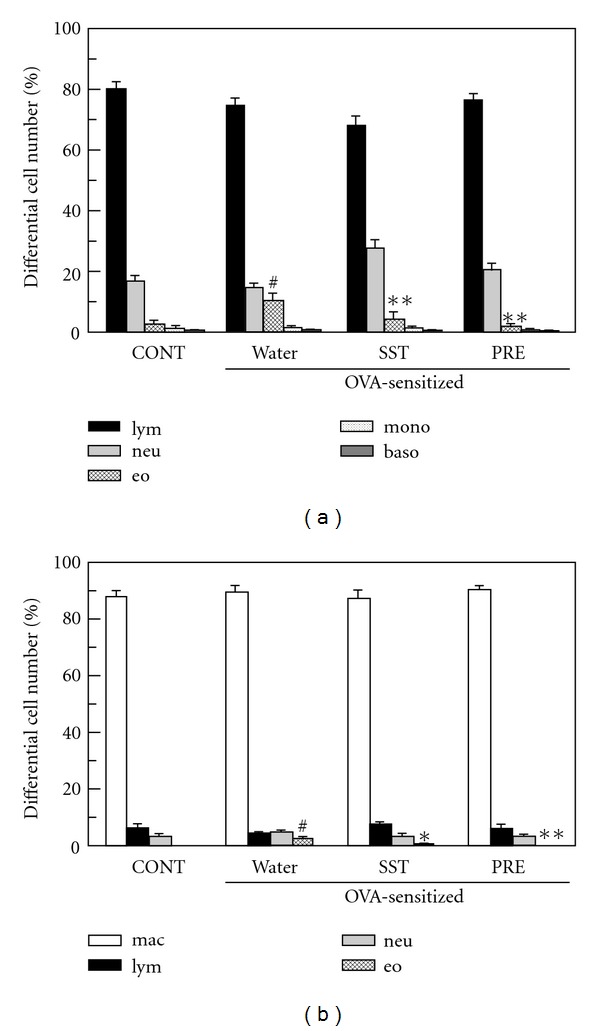
Effects of SST and prednisolone on differential cell number in blood and BAL fluid of OVAsensitized mice were measured. OVA-sensitized mice were treated with SST, prednisolone (PRE) or water as described in legend of Figure 2. Differential cell number in blood (a) and BAL fluid (b) were counted on 7 days after the OVA exposure. Each column represents the mean  ±  SEM of five to seven mice per group. ^#^ *P* < .0001 versus control; *P < .001 and **P < .0001 versus water/OVA-sensitized group (Tukey's test). mac (macrophage), lym (lymphocyte), neu (neutrophil), eo (eosinophil), baso (basophil), mono (monocyte).

The OVA-sensitized and OVA-challenged mice had significantly increased the eosinophil counts in blood and BAL fluid as compared to non-sensitized and OVA-challenged control mice (Figure 3). The percentage of eosinophils in blood and BAL fluid on lymphocyte count was also significantly elevated in OVA-sensitized and OVA-challenged mice (Figure 4). The administration of SST or prednisolone significantly reduced the eosinophil counts and percentage of eosinophils in blood (Figures 3(a) and 4(a)) and BAL fluid (Figures 3(b) and 4(b)) as compared to vehicle control. These results suggest that SST and prednisolone reduce the circulation of eosinophils in blood and infiltration of eosinophils in bronchoalveolar cavity of the OVA-sensitized and OVA-challenged mice.

### 3.3. Inhibition of Anti-OVA IgE Antibody Production by SST

**Figure 5:**
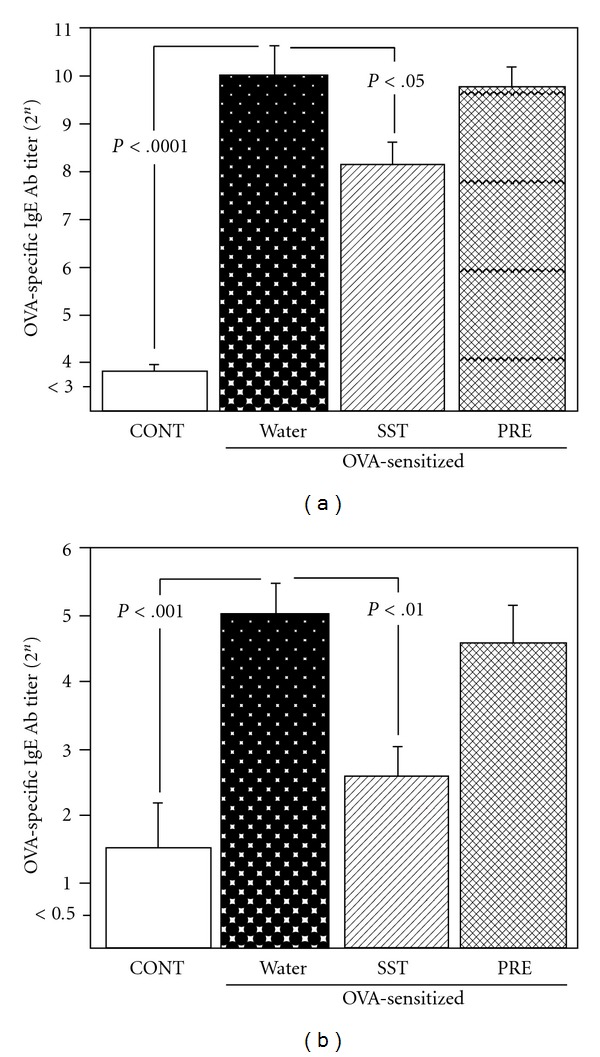
Effects of SST and prednisolone on OVA-specific IgE antibody titers in OVA-sensitized mice were measured. OVA-sensitized mice were treated with SST, prednisolone (PRE) or water as described in legend of Figure 2. OVA-specific IgE antibody titers in plasma (a) and BAL fluid (b) were determined at 7 days after OVA exposure. Each column represents the mean  ±  SEM of five to six mice per group. Statistical analysis was conducted using Tukey's test.

Effect of oral administration of SST on the anti-OVA IgE and IgG_1_ antibody titers was examined in the plasma and BAL fluid of OVA-sensitized mice as an airway inflammation model. Anti-OVA IgE antibody titers in the plasma and BAL fluid of the sensitized mice were significantly higher than those in the non-sensitized control at 7 days after the inhalation (Figure 5). Anti-OVA IgG_1_ antibody titers in the plasma and BAL fluid of OVA-sensitized mice were also significantly higher than those from control mice (data not shown). When SST or prednisolone was administered to the OVA-sensitized mice, anti-OVA IgG_1_ antibody titers in the plasma and BAL fluid were not affected at 7 days after the inhalation (data not shown). However, anti-OVA IgE antibody titers in the plasma and BAL fluid were decreased significantly by the administration of SST in comparison with that of water-administered sensitized mice at 7 days after the inhalation (Figure 5). Unexpectedly, prednisolone did not decrease the anti-OVA IgE antibody titers in comparison with that of water-administered sensitized mice at 7 days (Figure 5). These results suggest that SST decreases OVA-specific IgE antibody levels in BAL fluid and plasma, but prednisolone does not decrease the IgE antibody levels of the sensitized mice.

### 3.4. Reduction of AHR by SST

**Figure 6:**
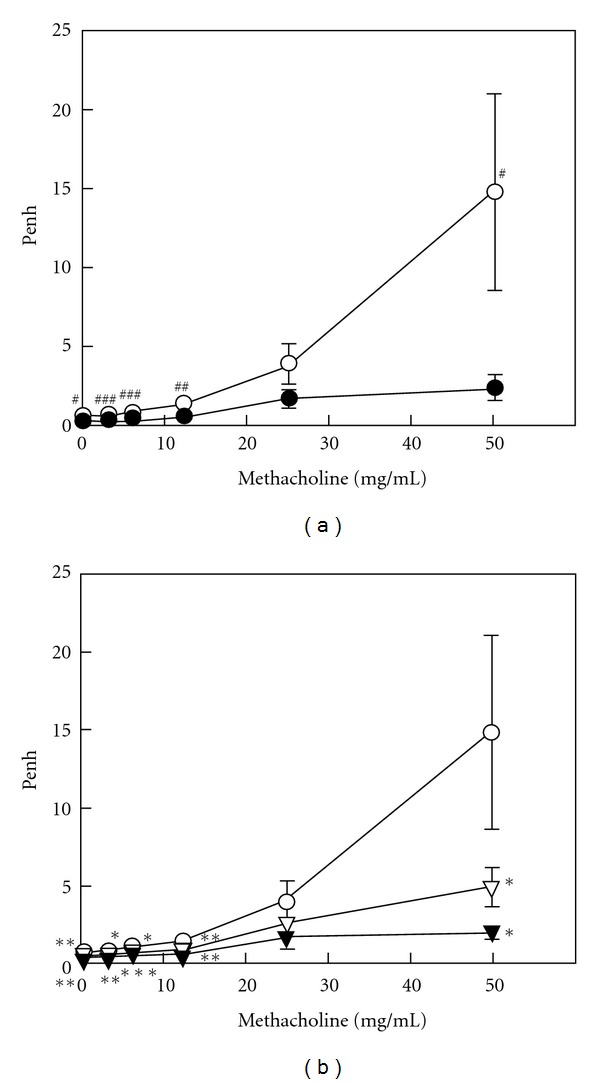
Effects of SST and prednisolone on airway hyperreactivity (AHR) in OVA-sensitized mice were measured with plethysmography. Non-sensitized mice were treated with water (closed circle), and OVA-sensitized mice were treated with SST (closed inverted triangle), prednisolone (open inverted triangle) or water (open circle) as described in legend of Figure 2. AHR to aerosolized methacholine (MeCh) was measured in unrestrained conscious mice at 1 h after the final treatment of drug. Mice were placed into the main chamber and were nebulized with PBS or MeCh (3.125, 6.25, 12.5, 25, 50 mg ml^−1^) for 3 min. Recording of Penh was taken for 5 min after each MeCh nebulization. Each bar represents the mean  ±  SEM of four mice per group. ^#^ *P* < .05, ^##^ *P* < .01 and ^###^ *P* < .001 versus water/non-sensitized group (Fisher's PLSD test). *P < .05, **P < .01 and ***P < .001 versus water/OVA-sensitized group (Dunnett's test).

The intraperitoneal sensitization and nebulized provocation of mice with OVA resulted in a dose-dependent increase in airway responsiveness to inhaled methacholine at 6 days after the inhalation (Figure 6). When SST or prednisolone was administered to the OVA-sensitized mice, the AHR was suppressed significantly at 6 days after the inhalation (Figure 6). These results suggest that SST and prednisolone decrease the AHR of the OVA-sensitized mice.

### 3.5. Two-dimensional Electrophoresis Analysis of Lung Tissue from OVA-sensitized Mice

**Figure 7:**
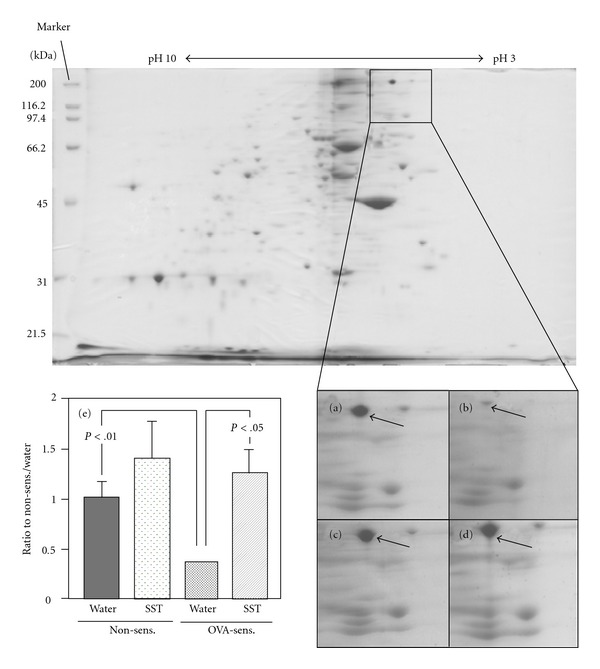
Effect of SST on protein expression in lung tissues of OVA-sentitized mice was measured by agarose 2D electrophoresis. OVA-sensitized or non-sensitized mouse was treated with SST or water as described in legend of Figure 2. Lung tissue was obtained at 7 day after the OVA exposure. Agarose 2D electrophoresis patterns of non-sensitized/water-treated mouse (a), OVA-sensitized/water-treated mouse (b), non-sensitized/SST-treated mouse (c) and OVA-sensitized/SST-treated mouse (d), and results of statistical analysis of spectrin *α*2 expression (e). Each column represents the mean  ±  SEM of four mice per group. Statistical analysis was conducted using Tukey's test.

The effect of SST extract was compared at the doses of 0.1 and 0.5 g kg^−1^ day^−1^ against OVA-specific IgE Ab titers in the airway inflammation in a mouse model. SST extract reduced the OVA-specific IgE antibody titers in BAL fluid significantly at 0.5 g kg^−1^ day^−1^ but not at 0.1 g kg^−1^ day^−1^ (unpublished data). Therefore proteomic analyses of lung tissues were performed from the mice administered SST extract at 0.5 g kg^−1^ day^−1^. Each lung tissue samples from non-sensitized and OVA-challenged treated with water vehicle (*n* = 4), OVA-sensitized and OVA-challenged treated with water vehicle (*n* = 4), non-sensitized and OVA-challenged treated with SST (*n* = 4), and OVA-sensitized and OVA-challenged treated with SST (*n* = 4) were compared protein spots each other by 2D electrophoresis. Among the all protein spots in 2D electrophoresis, OVA-sensitization (Figure 7(b)) altered the expression level of single protein species in lung tissue compared with non-sensitized mouse (Figures 7(a) and 7(e)). SST treatment did not affect the expression of protein on non-sensitized mouse (Figure 7(b)), but SST recovered the expression of protein level that was changed by OVA-sensitization in lung tissue (Figures 7(d) and 7(e)). This protein was identified as spectrin *α*2 by in-gel tryptic digestion of the protein followed by MS (database accession No. gi|51706071|ref|XP_207079.3; experimental MW, >200 kDa theoretical MW, 209 329 Da; SEQ score, 266.0; coverage, 17.2%).

### 3.6. Immunoblotting of Spectrin *α*2 in Lung

**Figure 8:**
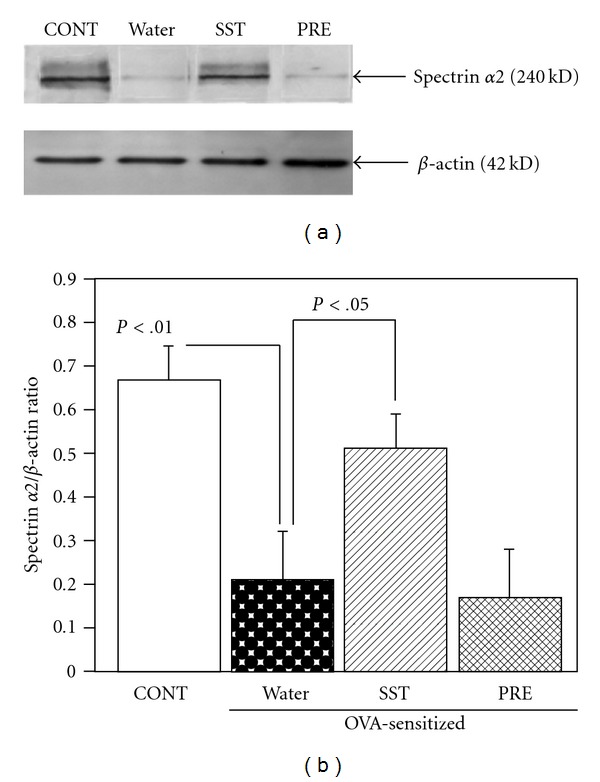
Effects of SST and prednisolone on spectrin *α*2 expression level in lung tissue of OVA-sensitized mice were measured by western blot analysis. OVA-sensitized mice were treated with SST, prednisolone (PRE) or water as described in legend of Figure 2. Lung tissue was obtained at 7 day after the OVA exposure. Western blot with *β*-actin was performed to verify that equivalent amounts of proteins were loaded in each lane. Western blotting patterns of spectrin *α*2 and *β*-actin (a), and results of statistical analysis of spectrin *α*2/*β*-actin ratio (b). Each column represents the mean  ±  SEM of five to seven mice per group. Statistical analysis was conducted using Tukey's test.

To verify the 2D electrophoresis findings, we conducted immunoblotting on a selected protein target. Western blotting of lung tissue using antibody targeted at spectrin *α*2 revealed that SST significantly up-regulated the reduced spectrin *α*2 level in OVA-sensitized mice (Figure 8). However, prednisolone did not up-regulate the expression of spectrin *α*2 in the lung tissue of OVA-sensitized mice (Figure 8).

### 3.7. Recovery of Spectrin *α*2 Expression in the Lung by SST

**Figure 9:**
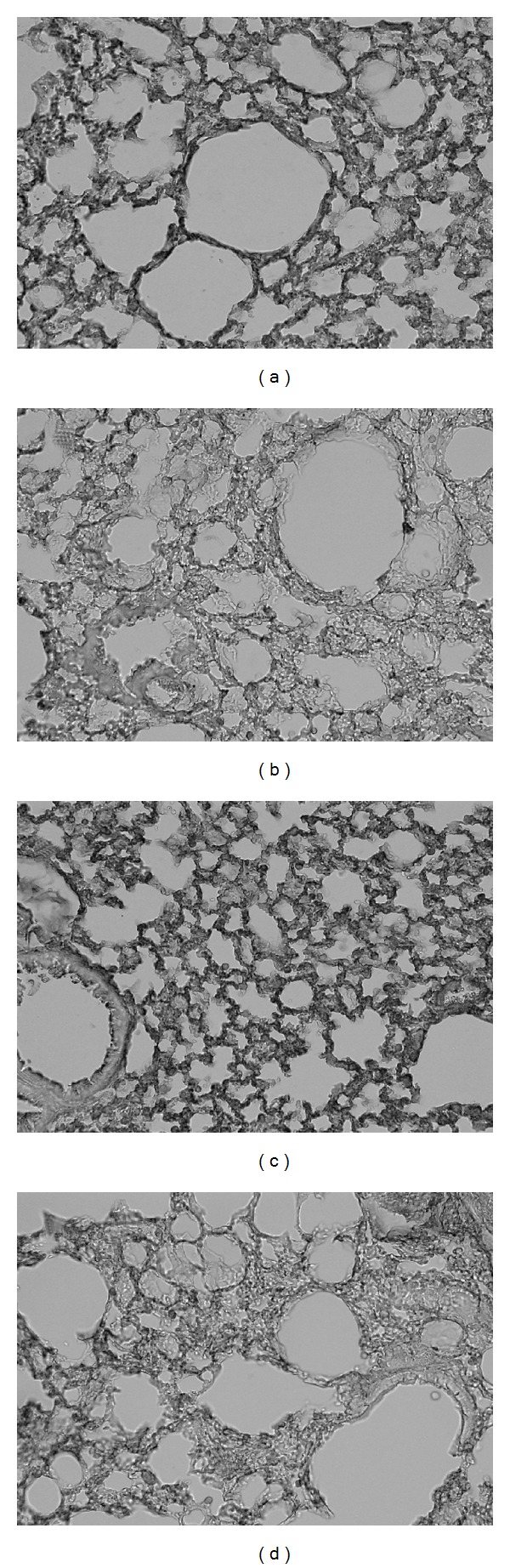
Effects of SST and prednisolone on spectrin a2 expression in lung of OVA-sensitized mice were measured by immunohistochemical staining. The spectrin a2 was stained with antispectrin a2 antibody. OVA-sensitized mice were treated with SST, prednisolone or water as described in legend of Figure 2. Lung tissue was obtained at 7 day after the OVA exposure. (a) Non-sensitized/water-treated mouse, (b) OVA-sensitized/watertreated mouse, (c) OVA-sensitized/SST-treated mouse and (d) OVA-sensitized/prednisolone-treated mouse. Magnification ×200.

Compared with non-sensitized mice (Figure 9(a)), OVA-sensitization resulted in the decrease of spectrin *α*2 expression in the type I alveolar epithelial cell membrane (Figure 9(b)), indicating that spectrin *α*2 expression in the type I alveolar epithelial cell membrane of airway inflammation model mice may be down-regulated by the sensitization of OVA. Administration of SST increased the expression of spectrin *α*2 (Figure 9(c)) as compared to the water-administered control (Figure 9(b)). However, administration of prednisolone did not recover the expression of spectrin *α*2 (Figure 9(d)).

## 4. Discussion

Bronchial asthma is a chronic inflammatory disease of the airways. Non-specific AHR, infiltration of inflammatory cells, and airway edema are the main features of bronchial asthma. The obstruction of the airway was also caused by hyperplasia of goblet cells and hypersecretion of mucus followed by dyspnea with wheezing, main features of bronchial asthma. Eosinophils are predominant among the inflammatory cells infiltrating the airways, and play a critical role in the pathogenesis of bronchial asthma. Thus, one therapeutic strategy for bronchial asthma would be to target the mechanisms involved in the accumulation and activation of eosinophils in the airways. Glucocorticosteroids are currently the most effective drugs available for treating the airway inflammation of asthma. Corticosteroid treatment reduces the number and activity of infiltrating inflammatory cells, particularly eosinophils, and damps microvascular leakage and other signs of inflammatory response [1, 35]. Glucocorticosteroids, however, induce significant systemic adverse effects when given for prolonged periods. Therefore, a safe alternative anti-asthmatic agent to corticosteroids is needed in the management of bronchial asthma.

**Figure 10:**
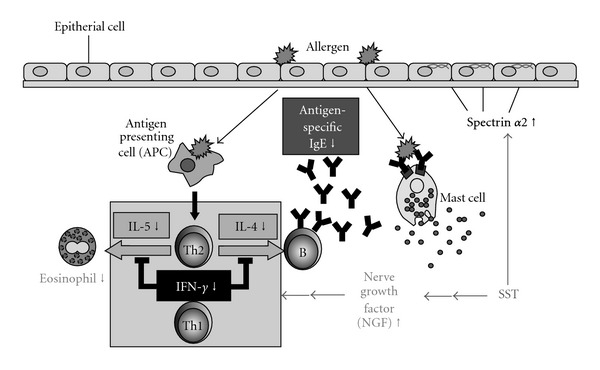
Hypothetical diagram of therapeutic effects of SST on airway inflammation.

SST has been widely used clinically for the treatment of allergic airway diseases including bronchial asthma and allergic rhinitis in Japan. The action mechanism of SST in allergic airway diseases has been studied *in vitro* and *in vivo*. However, its mode of action is still not fully elucidated for bronchial asthma. We have been reported SST reduced the OVA-specific IgE antibody titer in BAL fluids and the production of IL-4 and IL-5 in CD4^+^ T cells from lung of the OVA-induced allergic airway inflammation in a mouse model, and recovered the production of IFN-*γ* in the T cells [25, Figure 10]. In the present study, mode of action of SST was further studied using OVA-induced airway inflammation in a mouse model (Figure 10) and compared with a glucocorticosteroid, prednisolone.

Oral administration of SST reduced the infiltration of eosinophils, hypertrophy of smooth muscle and hyperplasia of goblet cells in lung tissue of OVA-induced airway inflammation in a mouse model (Figure 2). SST also significantly reduced the number of eosinophils in blood and BAL fluid of the mouse model (Figures 3 and 4). These results suggest that SST shows the anti-inflammatory effects in the respiratory tracts of bronchial asthma through the reduction of the number of eosinophils in blood and infiltration of eosinophils into bronchoalveolar cavity. It has been reported that various cytokines and chemokines, such as IL-5, IL-13 and eotaxin, and so forth. are related to the number and infiltration of eosinophils [4, 36]. Previously we have reported that SST reduced the production of IL-5 in CD4^+^ T cells from lung of the airway inflammation in a mouse model [25]. These results suggest that SST reduced the infiltration of eosinophils through the reduction of IL-5 in lung tissue. Study on effects of SST for other cytokines and chemokines are now in progress. SST significantly reduced the AHR of the airway inflammation in a mouse model (Figure 6). The AHR is a main future of bronchial asthma and causes the dyspnea against non-specific stimuli to respiratory tracts. SST may decrease symptoms in bronchial asthma patients through the reduction of AHR.

In present study, anti-OVA IgE antibody levels were reduced by oral administration of SST both in the plasma and BAL fluids of the airway inflammation model mouse (Figure 5). Omalizumab is a humanized monoclonal anti-IgE antibody recently approved for the treatment of allergic asthma, and inhibits allergic responses by binding to serum IgE, thus preventing their interactions with cellular IgE receptors [37]. The reduction of allergen-specific IgE antibody may be related to the efficacy of SST against bronchial asthma. In present study, anti-OVA IgG_1_ antibody levels were not affected by oral administration of SST both in the plasma and BAL fluids of the airway inflammation model mouse. It has been reported that serum IgG antibody can gain access to mucosal surfaces by passive diffusion [38]. Ito et al. [39] also reported that the serum IgG antibody move to the surface of the tracheal, bronchial and bronchiolar mucosa at a constant rate by diffusion. These results indicate that OVA-specific IgG_1_ antibody in BAL fluids may be originated from the serum OVA-specific IgG_1_ antibody. Therefore it was suggested that SST showed little effect against production of OVA-specific IgG_1_ antibody on humoral immune response.

Proteomic analysis with agarose 2D electrophoresis shows expression of spectrin *α*2, a high molecular weight protein (*∼*240 kDa), was down-regulated in the lung tissue of airway inflammation model mouse, and the expression was recovered by administration of SST (Figure 7). These results were confirmed by western blotting of lung tissues (Figure 8). Roh et al. [40] reported that proteomic analysis of lung tissues from OVA-induced airway inflammation in a mouse model, and the expressions of 11 spots were significantly changed in the mouse model compared with OVA-sensitized and non-challenged control at 48 h after the last OVA exposure. Whereas, the expression of one spot, spectrin *α*2, in lung tissues was significantly changed in the OVA-induced airway inflammation mouse model compared with non-sensitized control at 7 days after the last OVA exposure by proteomic analysis in present study (Figure 7). The difference may be caused by the duration from OVA-inhalation. Acute change of protein expressions observed at 48 h after the last OVA exposure may be subsided within 7 days. Immunohistochemical analysis of lung tissues indicates that the expression of spectrin *α*2 was reduced in the type I alveolar epithelial cell membrane of airway inflammation model mouse, and the expression was recovered by administration of SST (Figure 9). Spectrin, an *α*/*β* heterodimer, is one of the major components of the cortical cytoskeleton that lies beneath the plasma membrane [41]. It will be necessary to examine the relation among the expression of spectrin *α*2, bronchial asthma and pharmacological effects of SST in the future.

In present study, prednisolone, a corticosteroid, reduced the infiltration of eosinophils, hypertrophy of smooth muscle and hyperplasia of goblet cells in lung tissue of OVA-induced airway inflammation in a mouse model (Figure 2). Prednisolone also significantly reduced the number of eosinophils in blood and BAL fluid, and the airway hyperreactivity of the mouse model (Figures 3 and 4). These results indicate that prednisolone has a potent anti-inflammatory activity in present airway inflammation in a mouse model. SST significantly reduced the allergen-specific IgE antibody in BAL fluid and plasma (Figure 5), and recovered the down-regulation of spectrin *α*2 in the lung tissue in the mouse model (Figures 7–9). Nevertheless, prednisolone did not reduce the allergen-specific IgE antibody (Figure 5) and did not recover the expression of spectrin *α*2 (Figures 8 and 9). These results suggest that at least a part of action mechanism of SST against airway inflammation is different from that of prednisolone. Ikeda et al. reported that prednisolone reduced the OVA-specific IgE antibody in the serum in OVA-sensitized nasal allergy model mice [21]. In this experiment, oral administration of prednisolone was started at OVA-sensitization of the mice. In present study, prednisolone was orally administered only after the OVA-inhalation to the mice. These results indicate that prednisolone may be suppressed the OVA-sensitization step of the mice. Although most patients with asthma respond to corticosteroid treatment, small subsets of patients demonstrate persistent tissue inflammation despite treatment with high doses [42]. This corticosteroid resistance or failure results from interaction between susceptibility genes and the host's environment, in addition to immunologic factors [42]. SST may be effective for the treatment of the corticosteroid-resistant bronchial asthmatic patients through the different mechanism from corticosteroids. It will be necessary to examine the effectiveness of SST treatment for the corticosteroid-resistant bronchial asthma in the future.

In previous paper, we have reported that pinellic acid (9,12,13-trihydroxy-10*E*-octadecenoic acid) prepared from the tuber of *P. ternata* Breitenbach, one component of the herbs of SST [30], reduced the OVA-specific IgE antibody titer in the BAL fluids from the OVA-induced airway inflammation in a mouse model [25]. This result suggests the possibility that pinellic acid is related to the different mechanism between SST and prednisolone. Study on identification of active substance(s) from SST is now in progress.

## Funding

Grant-in-Aid for Scientific Research (C) (KAKENHI) from Japan Society for the Promotion of Science (JSPS) (17590601 for T.N.).
